# Cost effectiveness of optimal treatment of ADHD in Israel: a suggestion for national policy

**DOI:** 10.1186/s13561-019-0240-z

**Published:** 2019-07-09

**Authors:** Asher Ornoy, Avia Spivak

**Affiliations:** 1 Department of Medical Neurobiology, Hebrew University Hadassah Medical School, Jerusalem, Israel; 2 Department of Economics, Ben Gurion University, Beersheba, Israel

**Keywords:** ADHD, Adults, Behavior, Accidents, Crime, Substance abuse, Education, Cost effectiveness

## Abstract

**Objectives:**

There are well known behavioral complications of ADHD at adulthood such as learning difficulties resulting in lower education attainments; increased rate of car and other accidents; substance abuse; misconduct and imprisonment. These complications can be prevented or alleviated by effective treatment. In this study we calculated the economic burden of ADHD among adults in Israel and the cost of diagnosing and treating ADHD from childhood to adulthood. We then obtained the cost-benefit ratio of the treatment.

**Methods:**

The data were calculated using accepted estimations of prevalence and cost for the Israeli population assuming a prevalence of 4% among adults which is based on the ADHD prevalence among school age children.

**Results:**

The estimated cost per person with ADHD due to lower education attainment, higher involvement in crime and car accidents and more drug abuse is 289,969 USD and the estimated cost for optimal treatment is 41,667 USD. Hence, the benefit cost ratio is 7.02 and, assuming only 50% success of treatment, it is 3.51, still a very high cost benefit ratio.

**Conclusions:**

Since early diagnosis and appropriate treatment of ADHD is very effective in reducing the various symptoms and complications at adulthood thus enabling a better education and higher income, it seems important to diagnose and offer comprehensive treatment to children with ADHD. Moreover, it seems equally important to continue treatment at adulthood.

## Introduction

In the recent decades it has become clear that the sound development of adults depends much on the interaction of genetics and individual experience at childhood. In the words of Nobel Prize winner, the economist James Heckman: ‘Life cycle skill formation is a dynamic process in which early inputs strongly affect the productivity of later inputs… Four core concepts important to devising sound social policy toward early childhood have emerged from decades of independent research in economics, neuroscience, and developmental psychology… cognitive, linguistic, social, and emotional competencies are interdependent; all are shaped powerfully by the experiences of the developing child; and all contribute to success in the society at large [1].

The public policy implications of this insight are very costly. For example, if the vocabulary of children is determined at a very early age, high quality nursery schools are needed at a very young age [2]. The required teacher to student ratio is such that makes the type of intervention expensive. While we do not object to this kind of public policy – to the contrary – we offer in this paper a more economical and more modest intervention.

We propose to have national screening in Israel for ADHD at the elementary school system, and then treat the children with the disorder. Below we explain that this neurobehavioral disorder can harm the development of children thus impeding their full development. This may lead to lower incomes as adults, and to the pursuit of criminal careers for the misfits, as well as other harmful behaviors. We then proceed to estimate the benefits and the costs of this national program, and show its economic effectiveness with the tool of benefit-cost analysis.

### ADHD

Attention deficit hyperactivity disorder (ADHD) is a neurobehavioral disorder defined by the Diagnostic Statistical Manual 5 (DSM 5) as a “persistent disorder of inattention and/or hyperactivity –impulsivity that interferes with functioning or development” [3]. At childhood, ADHD seems to be more prevalent in males, but seemingly not at adulthood [4–6]. Diagnosis is usually made at school age, but in the more severe cases symptoms may be recognized earlier [7]. The prevalence of ADHD has increased significantly in the past decades, and it varies among populations; in school age children it ranges between 5 and 12% [4, 5].

In a study that compared the number of children living in the central regions of Israel aged 6–18 years and were prescribed methylphenidate in 2007 and 2011, the prevalence increased from 4.2% to 7.5% with 4 times more prescriptions among Jewish children as compared to Arab children [8].

We recently carried out a study on 971 Jewish children of grades 1 and 2 from 35 elementary schools in Jerusalem [9] and on 1225 Arab children in Kafr Kana using the DSM IV parents and teacher’s questionnaire. We found a prevalence of 9.5% among Jewish children and 7.35% among Arab children.

Although ADHD is a neurobehavioral disorder of childhood, in most cases it extends into- adolescence and in 40%-50% the symptoms also continue into adulthood [5, 10]. Children and adults with ADHD have a variety of psychiatric and other comorbidities, the more common being learning difficulties, oppositional defiant disorder (ODD), conduct disorder (CD), anxiety and depression [3, 5]. The severity of ADHD and the presence of psychiatric comorbidity, especially ODD and CD, are also important predictors for ADHD persistence into adulthood**.**

Many adults with ADHD suffer also from other long – term complications. The rate of abuse of alcohol and illicit drugs among adolescents or adults with ADHD is higher than in the general population tending to start earlier. ADHD persons are also more often involved in criminal acts, do not obey the laws and are more involved in accidents, especially in car and road accidents [11–14]. The more severe cases exhibit more of these complications.

#### Criminal acts, convictions and imprisonment

Dalsgaard et al. [12] found in a psychiatric clinic in Denmark among 206 adults with severe ADHD from childhood, that 47% of them had criminal convictions at adulthood, with a relative risk of 5.6 compared to those without ADHD. Within this group, males were two times more prone to a conviction than females. Mannuza et al. [15] studied 207 males with childhood ADHD at 18–25 years and found that 47% of them have been arrested as compared to only 14% in non-ADHD youth, and their crimes were more severe. Rosler et al. [16, 17] found that 45% of prison inmate males and 24.5% of females have ADHD and Einat and Einat [18] found in a randomly selected cohort of Israeli adults imprisoned that 57% had ADHD. Other investigators reported on similar findings [20, 21].

There are apparently no studies showing the benefit of early diagnosis and treatment of ADHD in direct reduction of crime and imprisonment, mainly because of the difficulties is planning such studies. However, several studies and reviews have demonstrated that early treatment of persons with ADHD significantly improved the social function and the antisocial behavior. ([19], Shaw et al).

It can therefore be summarized that the rate of criminal acts and imprisonment is at least 3 times higher among adults with ADHD as compared with those without ADHD.

Early diagnosis and treatment reduce the increased involvement in criminal acts.

#### Substance use disorder and substance abuse

substance use disorder (substance dependence, substance addiction) is defined as a condition where the use of illicit drugs causes physiological and behavioral symptoms. Substance abuse defines the repeated use of illicit drugs without dependence, but with social consequences.

Lee et al., found [22] that children with ADHD have at adulthood a 2–3-fold higher risk for nicotine abuse (OR 2.08), for Marijuana abuse (OR 2.78), for cocaine abuse (OR 2.05) and a general OR for illicit drugs dependence (substance use disorder) of 2.64 as compared with non-ADHD. Among adults with substance abuse, the rate of ADHD was found to be 15.2%, over 3 times the rate found in adults without substance abuse.

Dalsgaard et al. [23] found among 218 adolescents (183 boys and 25 girls) that the relative risk for substance abuse was 7.7. Early initiation of stimulant treatment (at childhood) decreased the risk of later substance abuse. Willens et al. [24] and Manuza et al. [25] reported on similar findings.

It can be summarized that the danger of substance abuse and substance use disorder among adolescents and adults with ADHD is 3–4 times higher and early diagnosis and treatment significantly reduces this danger.

#### Car accidents

More car accidents in persons with ADHD probably stem from the decreased concentration and the tendency not to follow rules and directions. Groom et al. [13] used the Driving Behavior Questionnaire and found that people with ADHD performed more violations of road rules and more car accidents. Barkley et al. found [26, 27] that 40% of adolescents with ADHD had at least 2 accidents by the age of 30, as opposed to only 6% in controls. Monetary damage was 3 times higher in ADHD than in non-ADHD. Similarly, increased rate of road accidents was described by other investigators [28, 29]. Pharmacological treatment was found to significantly decrease the rate of such accidents [13, 30–32].

It can be summarized that the rate of serious car accidents is at least doubled in drivers with ADHD compared to non ADHD drivers; early diagnosis and effective treatment almost normalizes that rate.

#### Learning difficulties

A high rate of learning difficulties (30%-50%) is observed in children with ADHD and a high rate of ADHD is present among children with learning difficulties and their scores were 10–15 points lower compared to their peers even when controlling for IQ. [33–35]. School dropout is proposed as the most important risk for children with ADHD, since lower education affects almost any facet of adult life, including long-term income and family settings**.** In a study on 370 children with ADHD compared to 740 non-ADHD children, Barbaresi et al. [36] found that the likelihood of ADHD children to drop out from high school is 2.7 times higher than in children without ADHD.

#### Educational achievements at adulthood

In a study by Mannuza et al. [25] on 91 adult men with ADHD compared to 95 controls, only 9% of those with ADHD had a Bachelor’s degree as opposed to 34% in the controls and only 1% had Master’s degree opposed to 6% in the controls. In a survey by Biederman and Faraone [4] on 500 adults with ADHD compared to 501 adults without ADHD in the US, the number of those with ADHD that did not finish high school was 2.5 times higher than controls. Hence, their income as adults was also significantly lower because of reduced work productivity. Kuriyan et al. [37] found that only 29.5% of young adults with ADHD completed 4 years of post-high school education as compared to 76.8% in controls without ADHD, and their unemployment rate was 6 times higher.

Barkley (34?) found that, 42% of children with ADHD are held back compared to only 13% in non-ADHD; 32–36% of adolescents do not complete high school compared to 5% in non-ADHD; only 22% enter college and 5% graduate. We found [9] that among 92, 7–9 years old children with ADHD in Jerusalem, 41% had learning difficulties.

Optimal treatment of ADHD children often alleviates the learning difficulties, mainly due to improved attention and short-term memory [33]. Ornoy et al. [38] found that among 27 school-age children with ADHD that received stimulant treatment the parents reported that learning abilities improved very much in 59%, significantly improved in other 33% and had no effect on learning only in 7% of the children. The children also received other supportive and educational treatments. Similar improvement in learning abilities by pharmacological treatment was demonstrated in adults by Lu et al. [39] as well as by other investigators. Improved educational achievements also have benefits in reducing crime rate and in decreasing accidents and drug dependence.

It can be summarized that the ability for high educational achievements of people with ADHD is reduced more than 50% compared to non – ADHD persons; early diagnosis and treatment increase these achievements.

All existing data are a description of relatively small cohorts of adults with ADHD. Large population studies, that seem to be lacking, might have changed these findings. We hope that studies like ours will raise the interest of decision makers and will result in detailed population studies. Such studies may fine tune our results.

The purpose of this study was to estimate the average cost of a non-treated person with ADHD taking into account the interference at adulthood in daily activities, their lower education, higher rate of substance abuse and involvement in accidents and crime. In addition we were interested to calculate the cost of effective treatment and the cost benefit ration.

## Methods

The cost of non-treated persons with ADHD is calculated in order to assess the economic benefits of early diagnosis at childhood and effective treatment throughout life. We presumed that comprehensive treatment is effective only in 40%-60% of ADHD cases, with an average of 50%. Calculation of economic gain from treatment was assigned both individually and also for the whole Israeli economy. We assumed, based on the prevalence of ADHD in school age children in Israel, that about 4% of adults have ADHD. The cost was calculated for each one of the complications individually. Medical expenses were calculated for 55 years, taking into account the average age at diagnosis (10 years) and the working years until retirement at 65. The loss of income was calculated for the ages of 25–65, which is the average age that people work and have an income.

Israel is one of the OECD countries and many of its economic features are common to the other OECD countries. Thus, the cost benefit of ADHD treatment in Israel may be relevant to many other countries.

We use in our calculations on the cost-benefit of successful treatment of ADHD the data on the different comorbidities assuming that their rate in the Israeli population of children and adults with ADHD are similar to those described in the literature. According to the Israeli Central Bureau of Statistics, there were in 2015 about 3,830,000 adults (1,942,000 women) aged 25–65, hence, 4% are about 153,000 people with ADHD. Our calculations are presented for an individual person, and the total cost for Israel is that received after multiplication with the presumed number of adults with ADHD.

Quantitative evaluation of the cost effectiveness was carried out using the method of Cost-Benefit analysis described by Mishan [40], in his classic book. Costs and benefits are calculated in terms of Israeli Shekel (NIS) translated to US Dollars (USD), with the rate of exchange for 2017 being 3.599 [41]. When the outcome has probability smaller than one, the procedure is to multiply by it, i.e. taking the expected value of the cost and the benefit. The costs and benefits are those incurred by society as a whole, including the ADHD patients as well as others. For example, the costs of accidents or crime include everything – from loss of income of the ADHD person to the loss of life of others and to the cost of the prison system.

The validity and usefulness of Cost-Benefit analysis is exhibited in the fact that governments in all parts of the world use it to evaluate public policies. In the US it began with a law from 1936 stipulating that all public projects must meet the criterion for surplus of benefits over costs. Since then, more areas have used Cost-Benefit analysis to evaluate public policy, to water management, environmental policies etc. In the academic world, Weisbord [42] has applied it to the treatment of mental illness, and Plotnic [43] to substance abuse.

Based on the studies mentioned above we estimate conservatively the usefulness of comprehensive treatment to be 50%. Hence, a benefit of treatment of only 50% was used for all our calculations, reducing the benefit/cost to half. The cost of treatment is an estimate, where some costs (i.e. the cost of diagnosis, of follow up and the drugs) are based on real cost and the other (parental guidance, psychological and educational support) are based on realistic assumptions.

## Results

### Cost of lower educational achievements

The educational data (Table 1) show the success of Israeli high school education: out of all employed persons, only 10.0% have less than 11–12 years of education. The 0–8 category includes mostly elderly people. About one third has 11–12 years, another third has 13–15, and the rest - 30.4% are academics – with 16+ years of education. The gross monthly income of academics is about double that of high school graduates – 3269 USD as compared to 1846 USD for the latter. This is known as the education premium; one of the recent known phenomena in Israel and abroad is its steep rise in the recent decades.

**Table 1:** Gross Income (USD) per Employee by Years of Schooling, 2011

	Number of years of schooling					
	Total	0–8	9-10	11–12	13–15	16+
Employees (thousands)	2590.50	103.8	155.6	856.8	685	787.8
Percent of population	100.0%	4.0%	6.0%	33.1%	26.4%	30.4%
Gross Income USD per month	2312	1488	1542	1746	2260	3269
Increase over last category			24.4%	13.2%	29.5%	44.6%
USD per hour work	13.65	7.45	9.0	10.14	13.64	19.22

Source: Table 22 of the Central Bureau of Statistics, Israeli Household Income Survey, 2011

Table 2 shows a comparison in the average income of a person with ADHD compared to a person without ADHD. We estimated the average percent of ADHD persons with a certain education on the basis of the data published regarding educational achievement of people with ADHD. The monthly difference in income is 310 USD and per year 3720 USD. The total (100%) benefit to income from education (education premium) for 40 years is thus **148,752 USD** (Table 2).

**Table 2:** Individual loss of income in people with ADHD in USD/month according to the percent with specific years of education*

Total	0–8	9-10	11–12	13–15	16+	
100%	4.0%	12.0%	55.6%	13.2%	15.2%	People with ADHD, %
7203	1488	1542	1746	2260	3269	income
8319	4.0%	6.0%	33.1%	26.4%	30.4%	non ADHD, %

The education premium per month is 310 USD, per year 3720 USD and per 40 years 148,800 USD

### Car accidents cost and its prevention

According to a report for the Ministry of Transportation [44], the total cost of accidents in Israel is 2.3% of the Gross Domestic Product (GDP). This is the cost to society as a whole: costs of injuries, deaths, physical damage and the cost of preventing authorities. The GDP of 2017 was 350.7 billion USD. The GDP per capita is projected to grow at an average rate of around 1.7% in the next decade. The long-term real interest rate today is between 0.5% and 1%. So, for the next decade, the present value of the cost is underestimated when using simple sum. For longer periods it is impossible to predict. The total cost of accidents per year is 2.3% of 350.7 = 8.07 billion USD per year. The number of drivers in 2017 is estimated as 4.007 million. Hence, the accident cost per year per driver is 2013 USD. Over 40 years of driving the cost is 80,520 USD.

ADHD persons are more liable to be involved in accidents, with a factor of 2.5, so the cost per person over 40 years is on average 201,300 USD**.** The difference **is 120,780 USD** (Table 3).

**Table 3:** Total Benefits from successful ADHD Treatment (per person, per life-time, USD)

Education premium loss	148,800
Car accidents prevention	120,780
Crime prevention	13,889
Drug abuse prevention	3500
Total	289,969

### Crime cost and its prevention

The conservative estimate of average life-career cost of a criminal in Israel is 166,660 USD [45]. It is based on the total cost of criminal actions to society - including the justice system, police, prisons, the costs of life lost in murders etc.- and to the income loss of incarcerated criminals.

Criminal actions are more prevalent among people with lower education, low socio-economic status and neglect. Untreated severe ADHD goes together with this profile. Out of all incarcerated criminals - about 50,000 people in Israel - about one-half suffer from ADHD. So, 25,000 out of 153,000 are criminals. Their probability is 1/6. The expected cost from not treating the individual is theoretically 27,778; for conservative reasons we take into account only 50% of this magnitude. Hence, **13,889 USD** (Table 3).

### Illicit drug dependence and its prevention

According to a report by the Israel antidrug authority from 2016, the number of drug addicts in Israel was 32,000 [46]. This is 0.84% of the adult population. The ADHD persons have 2.5 times higher probability for drug dependence meaning that 2.1% of them are addicted to illicit drug. The direct and indirect costs of life time drug dependence is estimated at 1 million NIS. It follows that preventing one ADHD person from being addicted has the benefit of (2.1%-0.84%)× 1,000,000 = **3500 USD** (Table 3).

### Economic benefit of treatment

Table 3 shows the total benefit of successful treatment of an individual with ADHD. The total is **356,381 USD.**

As stated in Table 4, the cost of optimal treatment during childhood and adulthood is for medication about 83 USD/month, 10 months/year it is 830 USD. Since medication is not taken throughout life, we calculated it for 27 years (about half of the 55 years), being 22,410 USD. We should, remember that people with ADHD need neurological or neuropsychiatric follow up at least once a year until adulthood and once in several years during adulthood. A visit for medical follow up in the public settings costs about 70 USD, and we presumed about 20 visits up to the age of 65, hence this is an additional cost of 1400 USD. Children and their parents may sometimes need psychosocial support. The cost for psychosocial support can be additional 840 USD for 20 h/year, generally no more than 4–5 years. This may maximally add additional **4200 USD** for each person for an average of 5 years of treatment. Parental guidance is generally given a few times, and very seldom more than 4 times. The total maximal cost for 4 years, is **2200 USD.**

**Table 4:** Total life-time estimated costs of full treatment (in USD per person)

	Estimated cost per first year/ USD	Estimated cost for the whole period/ USD (maximally 55 years)	
Diagnosis- one time	280	280	Once, at school age
Medical follow up	70	1400	20 times follow up
Medication	830 (10 months/year)	22,410	from diagnosis as needed, for 27 years
Psychological treatment (20 sessions, 42 USD/session	840 (20 sessions)	4200	as needed, maximally 5 repeats
Parental guidance (From first year, 55 USD/session)	550 (10 sessions)	2200	As needed, maximally 4 repeats
Educational support (one hour a week for 40 weeks, 30 USD/hour)	1200	10,800	Average of 9 years, to end of high school
Total	3770 USD	41,290	

### Benefit cost ration

Table 5 shows the benefit cost ration. For every NIS (or USD) invested in treating ADHD over the life time there is a gain of **7.02 USD**. If we assume only a success rate of 50% the return in **3.51 USD**. Besides the benefit to the patients, this high gain is making the early diagnosis and treatment of ADHD also very profitable economically. With higher success rate the benefit cost ratio goes up and with lower it goes down. For example, a success rate of 25% will cut the cost – benefit to 1.75. Even in this case, the treatment is warranted economically.

**Table 5:** Individual Benefit-Cost Ratio for persons with ADHD in USD

Total Benefits	289,969
Total Costs	41,290
Benefits to Costs ratio	7.02
Benefits to costs, assuming 50% success rate	3.51

The total benefit for Israel, assuming 153,000 adults with ADHD is **44.365** billion USD. The benefit per year (40 years) is **1.109** billion USD. The total annual cost would be about **112,819** million USD, gaining **996** million USD/year. Assuming a 50% success of treatment, it would be **498** million USD/year. A net profit of about half a billion dollars for a country with budgetary hardships and only 8 million people, is rather significant, not to add the social behavioral profits of effective treatment of ADHD.

## Discussion

It seems obvious that early diagnosis and successful treatment of ADHD in Israel are highly cost-effective as the economic benefit from treatment with 100% success is 7.02 and with 50% success it is 3.51. These ratios are robust to our assumptions, since we used conservative estimates throughout the study. The advantage of this study is the fact that the calculated figures were calculated bringing into consideration most daily life problems of adults with untreated ADHD. To our knowledge, this is the first study that considered most possible effects of untreated ADHD on daily life at adulthood. Note, however, that we did not consider the enormous social impact of untreated ADHD on the individual family and society, which have significant additional disadvantages and costs. This enhances the fact that we chose to be on the conservative side in our estimates of the economic advantage of treatment.

There are many studies that calculated the cost-effectiveness of ADHD treatment. Most of them, however, calculated the actual cost of different treatment modalities, concluding that the long acting stimulants are apparently more cost-effective than the short acting ones [47]. For example, Schlander et al. in Germany [48] calculated the cost of treating ADHD with a prevalence of 5% in children and 1.3% in adolescents. They found that the total direct medical cost for children with ADHD was 2.5 times higher than that for children without ADHD. This does not include additional indirect cost. Pelham et al. found [49] that the educational costs are higher by $4900 annually, compared to children without ADHD and the annual medical cost is $2636, with a total additional annual cost of $14,576. This also included the cost for the additional impact of higher rates of crime among those with ADHD. Due to the high prevalence of ADHD, and the fact that in about half of children it continues into adulthood, these costs impose a significant burden upon any economy. Our approach was to try and assess the benefit of treatment, calculating the gain following reduction of the costs on adults with ADHD by 50%.

Several investigators have calculated the loss in income of an untreated person with ADHD. Cohen [50] estimated in the US that the life-long monetary cost for the society of a child that does not graduate from high school is about $388,000. Doshi et al. [51] summarized the data of the annual cost in the US among adults with ADHD from 19 studies and found that the cost of productivity and income loss was 87–138 Billion dollars. Le et al. [52] estimated similarly the productivity losses of adults with ADHD in Europe and found that the annual cost is 143–339 Billion Euro. Both authors summarized their findings stating that this is a significant economic burden. We should emphasize that these estimates are close to our estimate of the cost of an untreated individual with ADHD to the Israeli society, which is **289,969 USD**. However, we also calculated the cost of optimal treatment, thus calculating the cost benefit ratio. It is obvious from our data that the highest contribution to the cost is the lower income that stems from the fact that children with ADHD have a lower education. We presume that good medical, educational and psychosocial supportive treatment, especially during childhood, can significantly improve school performance of the ADHD children, thus increasing their future level of education and income.

The lower education among children with ADHD which is the main contributor to the high cost, deserves some additional comments. Based on the literature and our data [38], we presumed that successful treatment also improves educational achievements. However, several studies have found that medical treatment indeed improved behavior, reduced the rate of substance abuse and of other complications of ADHD at adulthood but had a smaller influence on learning abilities [53]. Optimal success was achieved by combined educational and medical treatments as indeed suggested by the American Society of Child and Adolescent Psychiatry [54]. Moreover, learning difficulties warrant the use of specific educational treatment methods, thus pharmacological treatment may not be sufficient. Indeed, in our study, educational and psychosocial help were also offered to the children with ADHD [38].

Learning difficulties are influenced by genetic factors and a variety of other factors such as parental SES and behavior and cognitive level of the child and his parents. Moreover, the social difficulties that children with ADHD have in the regular school settings are generally not dealt with, increasing the learning difficulties. It is to be hoped that with increased awareness, the school environment will become more appropriate for all children with learning difficulties, including those with ADHD.

## Conclusion

The high benefit cost ratio, and the fact that early diagnosis of ADHD and treatment improves the outcome at adulthood, point to the importance of screening early school age children for ADHD. Furthermore, our estimates were carried out using conservative assumptions throughout the study. This screening can easily be done by the use of DSM 5 questionnaire or by other screening/diagnostic questionnaires filled out by the teachers, and if ADHD is suspected, also by the parents. This will enable the treating physicians to carry out proper diagnosis and offer appropriate treatment as early as possible.

## Data Availability

The detailed data on the economical calculations is available in Dr. Spivak’s office.

## Abbreviations

ADHD: Attention deficit hyperactivity disorder
CD: Conduct disorder
GDP: Gross Domestic Product
ODD: Oppositional defiant disorder
OECD: Organization for Economic Co-operation and Development
OR: Odds Ratio
USD: United States Dollars
